# Celiac Disease Deep Learning Image Classification Using Convolutional Neural Networks

**DOI:** 10.3390/jimaging10080200

**Published:** 2024-08-16

**Authors:** Joaquim Carreras

**Affiliations:** Department of Pathology, School of Medicine, Tokai University, 143 Shimokasuya, Isehara 259-1193, Japan; joaquim.carreras@tokai.ac.jp; Tel.: +81-463-93-1121; Fax: +81-463-91-1370

**Keywords:** artificial intelligence, convolutional neural network, computer vision, transfer learning, inflammatory bowel disease, celiac disease, machine learning, duodenum, inflammation, carcinoma

## Abstract

Celiac disease (CD) is a gluten-sensitive immune-mediated enteropathy. This proof-of-concept study used a convolutional neural network (CNN) to classify hematoxylin and eosin (H&E) CD histological images, normal small intestine control, and non-specified duodenal inflammation (7294, 11,642, and 5966 images, respectively). The trained network classified CD with high performance (accuracy 99.7%, precision 99.6%, recall 99.3%, F1-score 99.5%, and specificity 99.8%). Interestingly, when the same network (already trained for the 3 class images), analyzed duodenal adenocarcinoma (3723 images), the new images were classified as duodenal inflammation in 63.65%, small intestine control in 34.73%, and CD in 1.61% of the cases; and when the network was retrained using the 4 histological subtypes, the performance was above 99% for CD and 97% for adenocarcinoma. Finally, the model added 13,043 images of Crohn’s disease to include other inflammatory bowel diseases; a comparison between different CNN architectures was performed, and the gradient-weighted class activation mapping (Grad-CAM) technique was used to understand why the deep learning network made its classification decisions. In conclusion, the CNN-based deep neural system classified 5 diagnoses with high performance. Narrow artificial intelligence (AI) is designed to perform tasks that typically require human intelligence, but it operates within limited constraints and is task-specific.

## 1. Introduction

Celiac disease (CD) is a gluten-sensitive immune-mediated enteropathy that occurs in genetically predisposed individuals [1]. Diagnosis of celiac disease is made by combining clinical data, serological tests, and histopathological features [1,2]. Although celiac disease is a disease of infants, its onset usually occurs in patients aged between 10 and 40 years, when the typical signs of malabsorption are often replaced by an atypical presentation [3,4,5,6].

The clinical presentation is variable and exhibits a continuum spectra [3,4,5,6], with several degrees of severity correlated with histological severity and levels of tissue transglutaminase [7,8]. The “classical” gastrointestinal symptoms include persistent diarrhea, abdominal distension, weight loss, abdominal pain, constipation, and vomiting [9]. Celiac disease is also associated with several non-gastrointestinal manifestations, such as growth and development alterations, neurologic and behavioral symptoms, liver disease, iron deficiency, skin alterations (dermatitis herpetiformis), dental and metabolic bone diseases, arthritis, and cardiomyopathy [9,10].

Histological characteristics of the small intestine (usually evaluated using duodenum biopsy) include mucosal inflammation, villous atrophy, and crypt hyperplasia that occur after exposure to dietary gluten; signs that improve after removing gluten from the diet [11]. These histological features are variable and range from mild alteration with only increased numbers of intraepithelial lymphocytes, to severe atrophy and epithelial apoptosis [12,13,14,15,16]. These alterations are assessed in several classifications, including the Marsh [17], Marsh–Oberhuber [18], Corazza–Villanacci [19], Q-Marsh scale [20], and Q-histology [2].

The pathogenesis of celiac disease includes genetic factors (HLA DR3-DQ2, DR4-DQ8, several non-HLA loci, and autoimmune disorders), adaptive immune response (gliadin reactive T lymphocytes), autoantibodies and intraepithelial lymphocytes (IELs), and innate immune response. In patients with celiac disease, the immune response to fractions of gliadin results in an abnormal inflammatory reaction characterized by infiltration of the lamina propria and epithelium by chronic inflammatory cells and villous atrophy [4]. A comprehensive review of the pathogenesis was conducted in our recent publication [21].

Primary treatment for celiac disease is a gluten-free diet. Persistent or recurring symptoms may be due to a lack of adherence to dietary protocol, an incorrect initial diagnosis, or complications of refractory celiac disease and lymphoma [1]. Among the different primary intestinal T-cell lymphomas, enteropathy-associated T-cell lymphoma (EATL) [22,23,24,25] may be preceded by refractory celiac disease [26].

The diagnosis of celiac disease is based on the combination of clinical data (enterologist), serology (clinical pathologist), and duodenal biopsy with histological evaluation performed by a certified anatomical pathologist [1]. Artificial intelligence technology allows computers to imitate human intellectual capacity and solve problems [27]. Modern computer vision systems exhibit extraordinary image recognition and analysis accuracy. However, these systems do not understand what they observe. In recent years, applications of artificial intelligence in celiac disease diagnosis have been developed. The most relevant studies can be divided into two groups, the ones that perform computer vision on endoscopic images and others that performed histological analyses [28,29,30,31,32,33]. These studies have provided the basis for further development of the analysis of celiac disease using artificial intelligence, such as the introduction of other types of pathologies as shown in our study.

Several machine learning and deep learning algorithms have been developed to construct models that make predictions on images. Convolutional neural networks are supervised algorithms that are mostly used for image recognition workloads [34]. Top pre-trained models for image classification are the following: ResNet (Residual Networks), Inception (GoogLeNet), VGG (Visual Geometry Group), EfficientNet, DenseNet (Dense Convolutional Network), MobileNet, NASNet (Neural Architecture Search Network), Xception (Extreme Inception), AlexNet, and Vision Transformers (ViT).

This study used a convolutional neural network to classify images of celiac disease, small intestine control, duodenal inflammation, duodenal adenocarcinoma, and Crohn’s disease.

## 2. Materials and Methods

**Figure 1:**
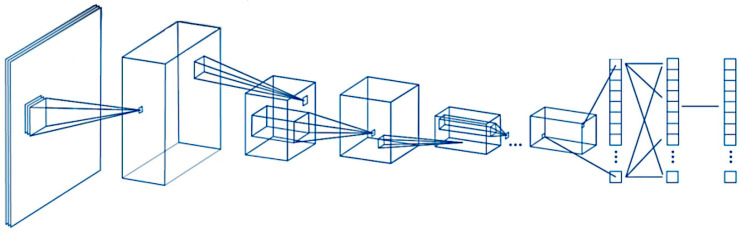
General design of the convolutional neural network. A convolutional neural network (CNN) is a deep learning algorithm that takes an input image, assigns weights/biases to different components of the image, and classifies all the images. There are three major components of the network: the convolutional layer, the pooling layer, and the fully connected layer.

**Figure 2:**
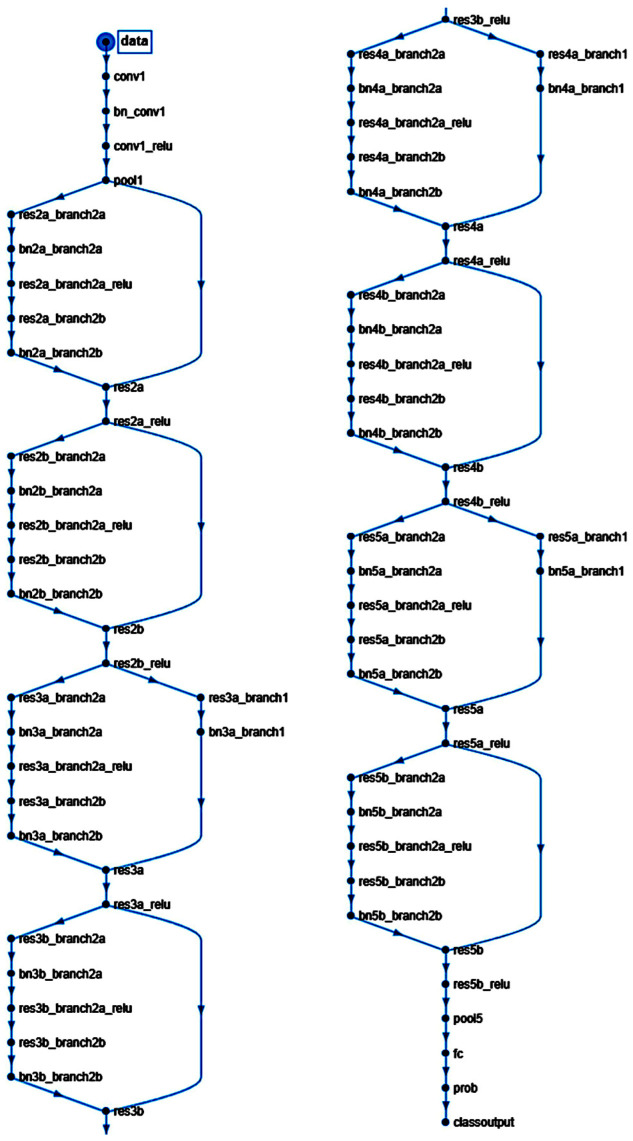
Structure of the convolutional neural network of this study (based on ResNet-18).

A script was written to create and train a deep learning network with 71 layers and 78 connections (Figure 1 and Figure 2). The script was run to create network layers (Appendix B), import training and validation data, and train the network. The code was created in MATLAB (R2023b Update 8 (23.2.0.25999560) 64-bit (win64) 29 April 2024) (MathWorks, Tokyo, Japan) and was based on transfer learning from the ResNet-18 (version 23.2.0) [35] (Figure 1 and Figure 2). All analyses were performed using a desktop computer equipped with an AMD Ryzen 9 7950X CPU (AMD Japan Ltd., Marunouchi, Chiyoda-ku, Tokyo, Japan) [36], 32 Gb of RAM, and an Nvidia GeForce RTX 4080 super-graphics card (Nvidia, Minato-ku, Tokyo, Japan) [37].

ResNet-18 is a pre-trained model that was previously trained in a subset of images in the ImageNet database [38]. This database includes 1000 types of objects and contains more than 1,000,000 images. ResNet-18 is a convolutional neural network with 18 layers. The input size is 224-by-224 (224 × 224 × 3). Size: 44.0 MB. Parameters: 11.7 M.

The analysis of the convolutional neural network (CNN) included the following steps: loading the pre-trained network, replacement of final layers, training of the network, prediction and assessment of network accuracy, and deployment of results.

**Figure 3:**
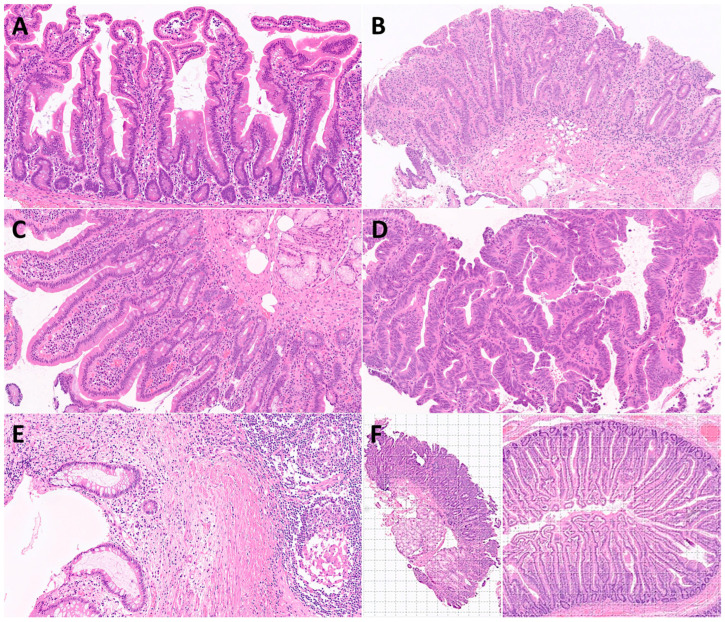
Characteristic histological images of the small intestine (duodenum). Duodenal control (**A**); celiac disease (**B**); inflammatory duodenum (**C**); duodenal adenocarcinoma (**D**); Cronh’s disease (**E**); examples of image cropping at 224-by-224 (224 × 224 × 3) size (F).

The input images were hematoxylin and eosin (H&E) stainings of several diseases (Figure 3).

The diagnostic dataset included hematoxylin and eosin (H&E) staining of 16 celiac disease patients (57 biopsies), selected from the Department of Pathology, Hospital Clinic of Barcelona, Spain, as previously described [21]. The clinicopathological characteristics, such as age, sex, biopsy location, anatomical pathology diagnosis, and the Marsh–Oberhuber histological grade [21,39,40] are shown in Appendix A.

**Figure 4:**
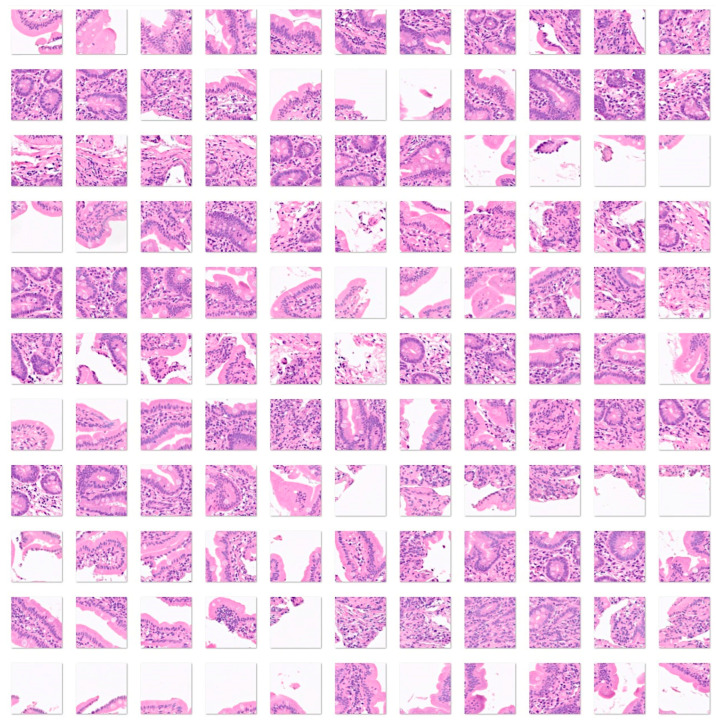
Images of celiac disease. Celiac disease is a gluten-sensitive immune-mediated enteropathy that occurs in genetically predisposed individuals. Diagnosis of celiac disease is made by combining clinical data, serological tests, and histopathological features. Celiac disease is characterized by mucosal inflammation, intraepithelial lymphocytosis, chronic inflammation in the lamina propria, villous atrophy, and crypt hyperplasia. When the disease persists despite the gluten-free diet, it is called refractory celiac disease, type I (the intraepithelial lymphocytes display normal phenotype, associated with good prognosis when combined with immunosuppressive therapy) and type II (loss of phenotype of the intraepithelial lymphocytes, monoclonal rearrangement of T-cell receptor, and higher risk of developing enteropathy-associated T-cell lymphoma (EATL)) [41,42,43,44,45,46,47,48,49]. The input size is 224-by-224 (224 × 224 × 3). Original magnification 200×.

**Figure 5:**
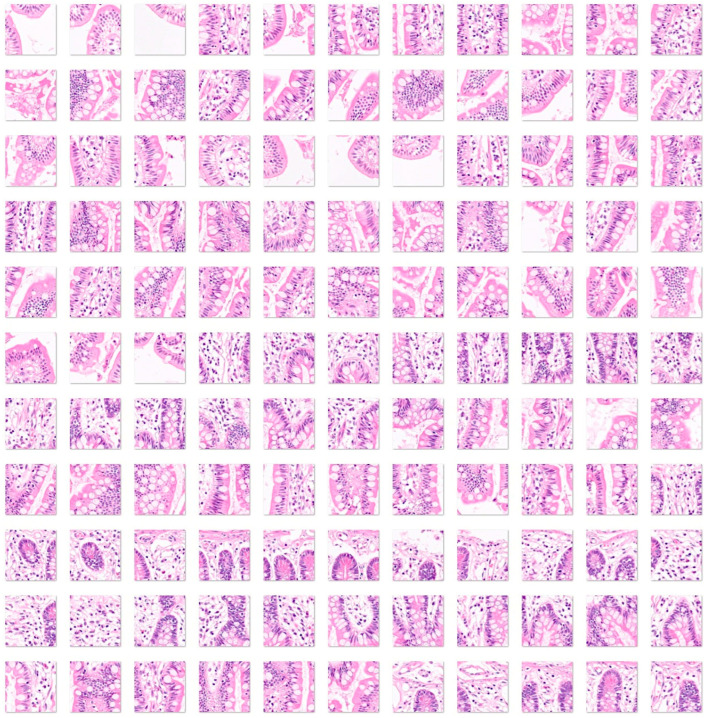
Images of small intestine control. The small intestine is located between the stomach and the large intestine. It is comprised of three parts: the duodenum, jejunum, and ileum. The histological structure has four main layers. The mucosa is the innermost layer that contains epithelium, lamina propria, and muscularis mucosae. The submucosa is a connective tissue layer with blood vessels, lymphatics, and the submucosal plexus. The muscularis externa contains two smooth muscle layers, the inner circular and the outer longitudinal layer. Between them, the myenteric plexus is found. The outermost layer is the adventitia comprised of fibroblasts, collagen, vessels, and nerves. The adventitia is covered by mesothelium (serosa). The small intestine is characterized by high absorption. The mucosa and submucosa form large folds (plicae) in circular manner. The plicae contain microvilli. The epithelium has several components, enterocytes, globet cells, crypts of Lieberkuhm, enteroendocrine cells, and Paneth cells [50,51]. This figure shows images of the ileum. Additionally, images obtained from the duodenum were included in the dataset. The input size is 224-by-224 (224 × 224 × 3). Original magnification 200×.

First, the input data for celiac disease included 7294 images, and the small intestine control included 11,642 images. The color images had three channels: red, green, and blue. An example is shown in Figure 4 and Figure 5.

The data (images) were split into three sets: a training set used for training the network (70%), a validation set used for testing its performance as it was trained (10%), and a test set (holdout) used after training to assess how well the network performed on new data (20%). The order of the images was randomized to ensure that the network learned the classes at a more even rate. As transfer learning (adjustment of a pre-trained network) was performed on ResNet-18, the fully connected and classification layers were removed and replaced with new layers with an output size of 2. Augmentation was not performed during training. To avoid overfitting, the initial learning rate was set to 0.001. The number of maximum epochs was set to five.

Data normalization was applied to the input images: imageInputLayer (an image input layer inputs 2-D images to a neural network and applies data normalization), and batchNormalizationLayer (a batch normalization layer normalizes a mini-batch of data across all observations for each channel independently. To speed up training of the convolutional neural network and reduce the sensitivity to network initialization, batch normalization layers are used between convolutional layers and nonlinearities, such as ReLU layers. Layer = batchNormalizationLayer (Name, Value) creates a batch normalization layer and sets the optional TrainedMean, TrainedVariance, Epsilon, Parameters and Initialization, Learning Rate and Regularization, and Name properties using one or more name-value pairs. After normalization, the layer scales the input with a learnable scale factor γ and shifts it by a learnable offset β) [52].

**Figure 6:**
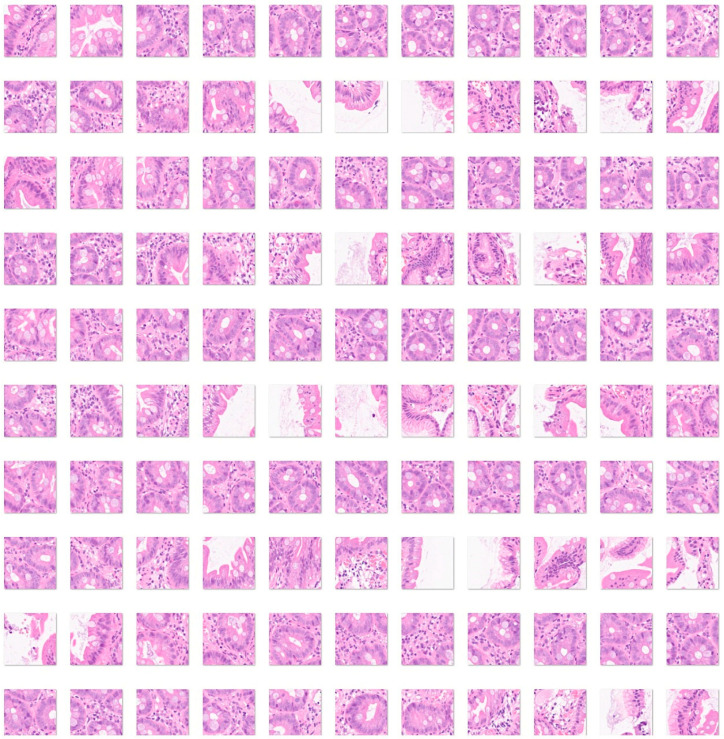
Images of nonspecific inflammatory small intestine. The input size is 224-by-224 (224 × 224 × 3). Original magnification 200×.

Second, the analysis was repeated by adding a third histological subtype of nonspecific inflammation of the small intestine (duodenum). Therefore, in this analysis, the input data included 7294 images of celiac disease, 11,642 images of small intestine control, and 5966 images of the small intestine (duodenum) with chronic and acute inflammation (Figure 6).

**Figure 7:**
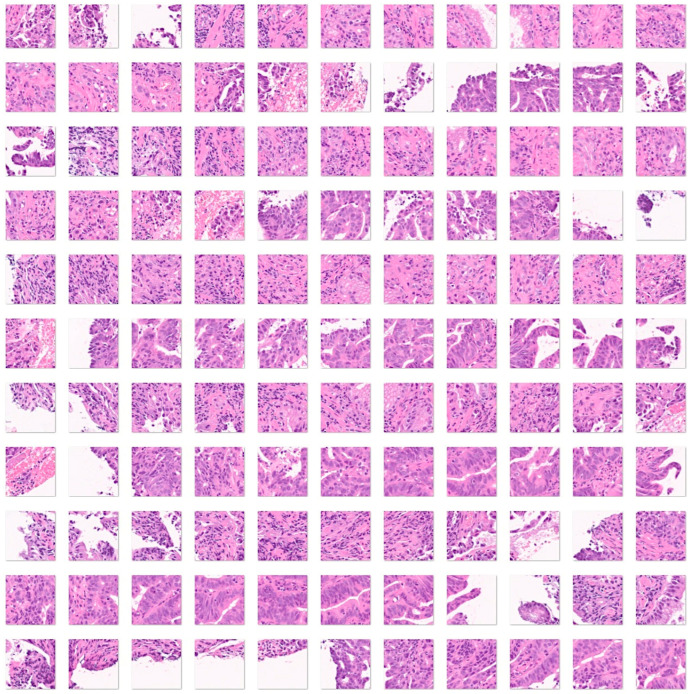
Images of duodenal adenocarcinoma. Small bowel adenocarcinomas are histologically very similar to colorectal adenocarcinomas. Adenocarcinomas are characterized by columnar epithelial cells with elongated and pseudostratified nuclei that form a complex glandular structure. There is nuclear polymorphism, loss of epithelial polarity, and necrosis. Inflammatory, autoimmune, genetic, and familiar diseases are common risk factors, including celiac disease, Crohn’s disease, familiar adenomatous polyposis, Peutz–Jeghers syndrome, and Lynch syndrome [53,54,55,56,57]. The input size is 224-by-224 (224 × 224 × 3). Original magnification 200×.

Third, a fourth histological subtype of 3723 images of duodenal adenocarcinoma (Figure 7) was added as test images of the previously trained convolutional neural network. The purpose of this analysis was to determine how the previously trained network, which was trained using celiac disease, small intestine control, and non-specific inflammation of the duodenum, could classify an unknown histological disease.

Fourth, a convolutional neural network was trained, including as input all the histological subtypes of celiac disease, small intestine control (both duodenum and ileum), non-specific inflammation of the duodenum, and duodenal adenocarcinoma (Figure 4, Figure 5, Figure 6 and Figure 7).

**Figure 8:**
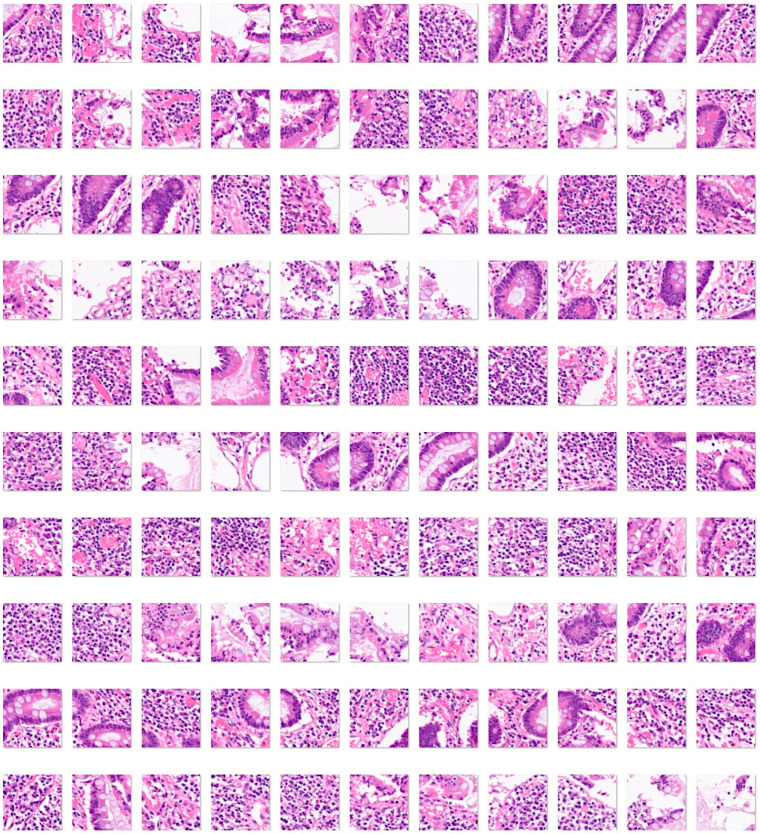
Images of Crohn’s disease. Crohn’s disease is an idiopathic chronic inflammatory condition that can affect both the upper and lower gastrointestinal tract, but usually involves the distal ileum and proximal large intestine. The diagnostic criteria are segmental disease, transmural inflammation, noncaseating granulomas, deep fissuring ulcers, and ileal involvement [58,59,60]. The input size is 224-by-224 (224 × 224 × 3). Original magnification 200×.

Finally, to expand the diagnosis differential into other intestinal pathologies related to alterations to the immune tolerance and immune homeostasis of the gut, the model included 13,032 images of Crohn’s disease (Figure 8).

All cropped images of 224-by-224 (224 × 224 × 3) size were reviewed by the histopathologist (J.C.) and non-diagnostic and artefactual images were excluded from the datasets.

## 3. Results

### 3.1. Celiac Disease vs. Small Intestine Control

The data (images) were split into three sets: a training set used for training the network (70%), a validation set used for testing its performance as it was trained (10%), and a test set used after training to assess how well the network performed on new data (20%).

**Figure 9:**
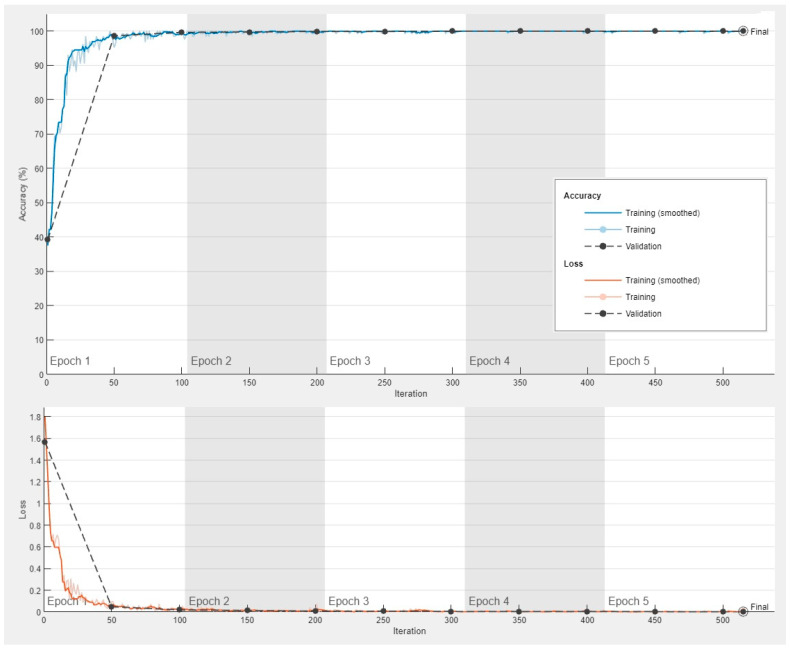
Training progress of the convolutional neural network for the classification of celiac disease and small intestine control.

The progress of the convolutional neural network (ResNet-18) was satisfactory with a high validation accuracy. The training cycle included 5 epochs, 515 iterations, and 103 iterations per epoch. The validation cycle included 50 iterations. Within the first 100 iterations, the accuracy percentage reached nearly 100%, and the loss the 0 value (Figure 9).

**Figure 10:**
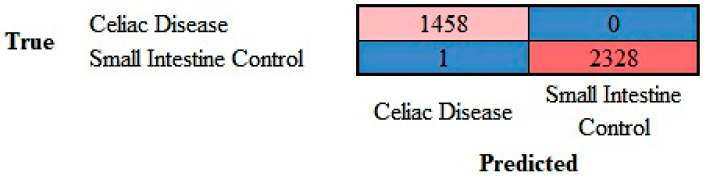
Confusion matrix of celiac disease and small intestine control. This image shows the confusion matrix of the test set, which includes the analysis of images not previously used in the training and validation steps (i.e., the holdout data). The accuracy of predicting celiac disease was 99.97%.

The images in the test set (20%) were classified by the trained network. The results are shown as a confusion matrix (Figure 10). The performance parameters for celiac disease were as follows: accuracy, 99.97%; precision, 99.93%; recall, 100%; F1-score, 99.97%; specificity, 100%; and false positive rate (FPR), 0.04% (Table 1).

### 3.2. Comparion between ResNet-18 and Other Top Models for Image Classification in the Celiac Disease vs. Small Intestine Control

**Figure 11:**
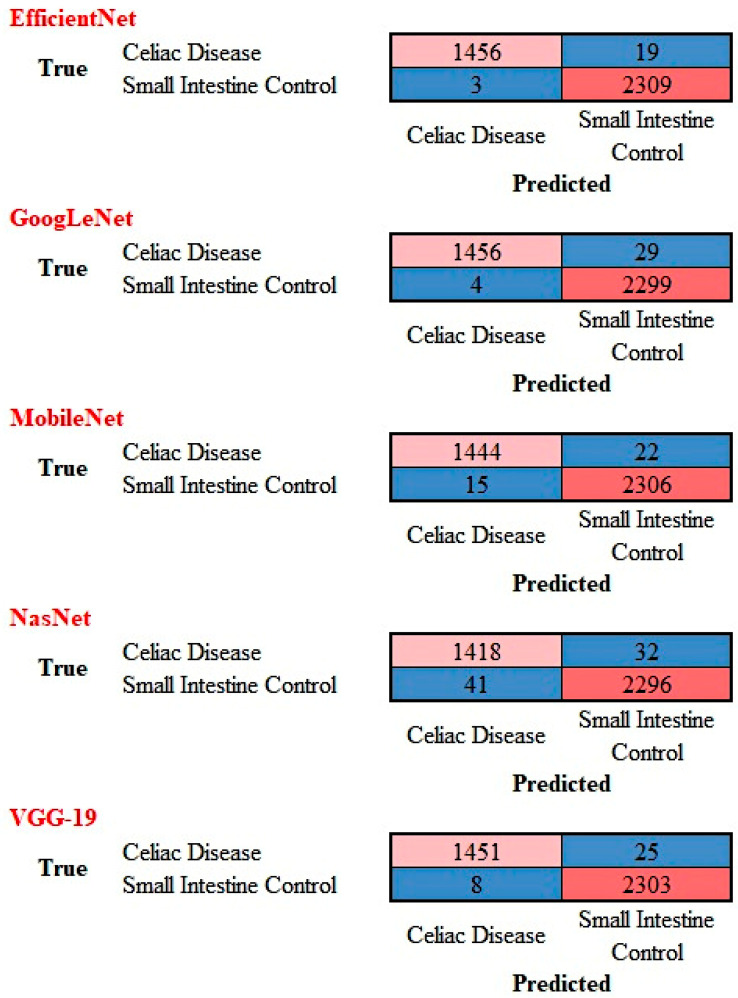
Confusion matrix of celiac disease and small intestine control using different AI models. This image shows the confusion matrices of the test set, which includes the analysis of images not previously used in the training and validation steps (i.e., the holdout data).

The performance of ResNet-18 was compared with other top pre-trained models for image classification including GoogLeNet, VGG-19, EfficientNet, MobileNet, and NASNet. The confusion matrices are shown in Figure 11 and the performance parameters in Table 2. Overall, these models also classified the images with good performance, although the accuracy was slightly lower.

### 3.3. Celiac Disease vs. Small Intestine Control vs. Duodenal Inflammation

**Figure 12:**
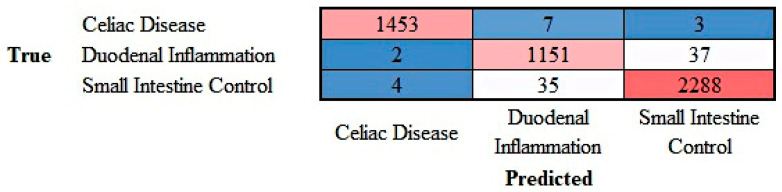
Confusion matrix of celiac disease, duodenal inflammation, and small intestine control. This image shows the confusion matrix of the test set, which includes the analysis of images not previously used in the training and validation steps (i.e., the holdout data).

The progress of the training of the convolutional neural network (ResNet-18) is shown in Appendix C. The results are shown as a confusion matrix (Figure 12).

The class-wise performance is summarized in Table 3.

### 3.4. Test for Duodenal Adenocarcinoma on Previously Trained Network

Images of duodenal adenocarcinoma were tested directly on the previously trained ResNet-18 network that had classified celiac disease, small intestine control, and duodenal inflammation. The analysis showed that the convolutional network classified duodenal adenocarcinoma as duodenal inflammation in 63.65% of images, small intestine control in 34.73%, and celiac disease in 1.61% of images. Therefore, a previously trained network can classify an unknown type of image but incorrectly diagnoses the image.

### 3.5. Celiac Disease vs. Small Intestine Control vs. Duodenal Inflammation vs. Duodenal Adenocarcinoma

**Figure 13:**
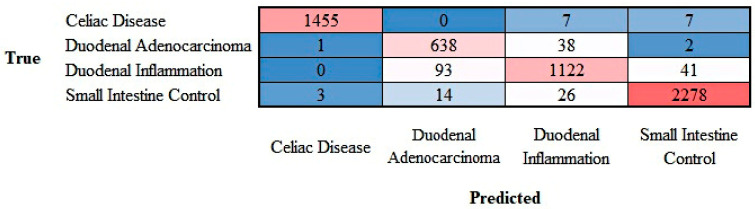
Confusion matrix of celiac disease, duodenal adenocarcinoma, duodenal inflammation, and small intestine control. This image shows the confusion matrix of the test set, which includes the analysis of images not previously used in the training and validation steps (i.e., the holdout data).

The progress of training the convolutional neural network (ResNet-18) is shown in Appendix D. The results are shown as a confusion matrix (Figure 13).

The class-wise performance is summarized in Table 4.

### 3.6. Addition of Crohn’s Disease into the Dataset

**Figure 14:**
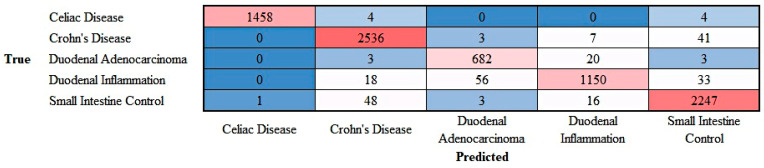
Confusion matrix of celiac disease, Crohn’s disease, duodenal adenocarcinoma, duodenal inflammation, and small intestine control. This image shows the confusion matrix of the test set, which includes the analysis of images not previously used in the training and validation steps (i.e., the holdout data).

To expand the analysis into other pathologies, Crohn’s disease images were added into the dataset. Figure 14 shows the confusion matrix with 5 classes that includes celiac disease, Crohn’s disease, duodenal adenocarcinoma, duodenal inflammation, and small intestine control. The overall accuracy of the test set was 96.87% The performances of each diagnosis is shown in Table 5. The output is shown in the Supplementary Material.

### 3.7. Grad-CAM Analysis

**Figure 15:**
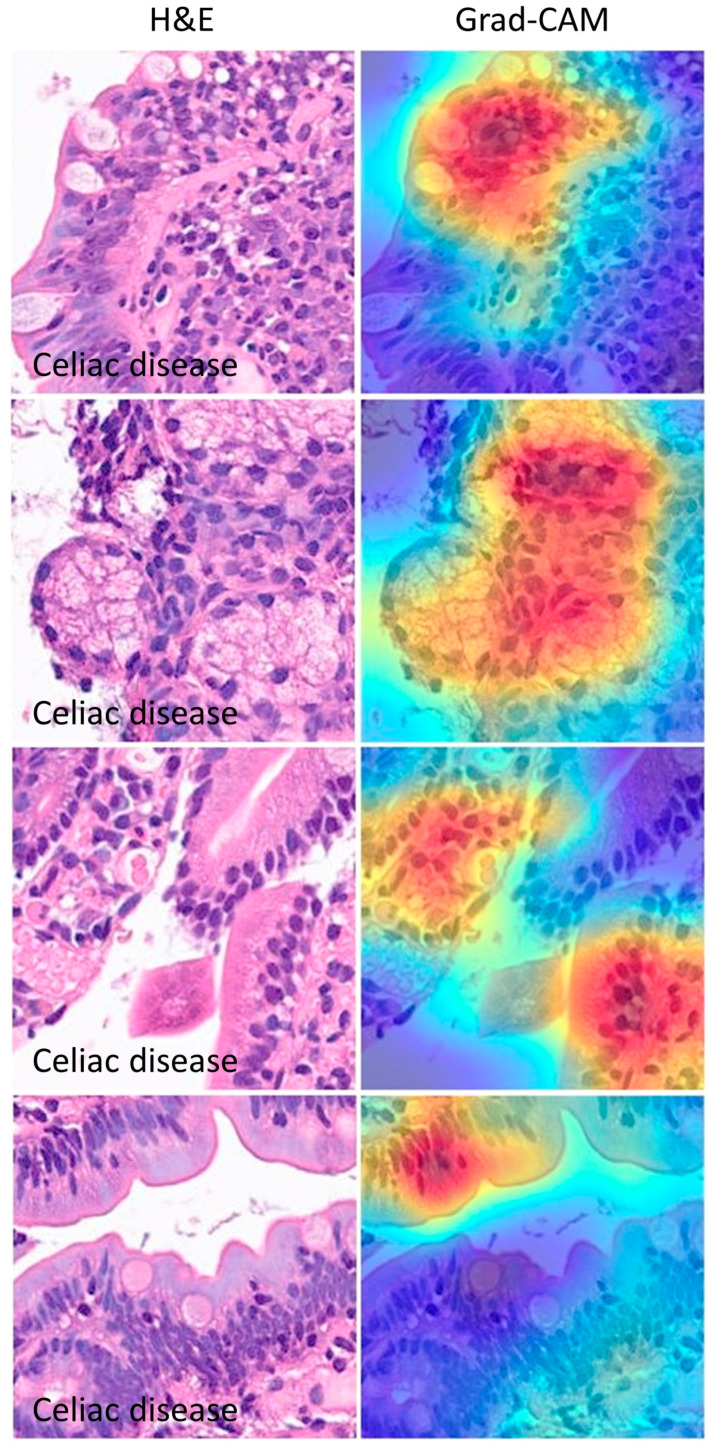
Grad-CAM images of celiac disease. The gradient-weighted class activation mapping (Grad-CAM) visualizes which parts of an image are important to the classification decision of a network. From the histopathological point of view, the Grad-CAM showed that the CNN was focusing on the important parts of the tissue such as the epithelial layer and inflammation.

**Figure 16:**
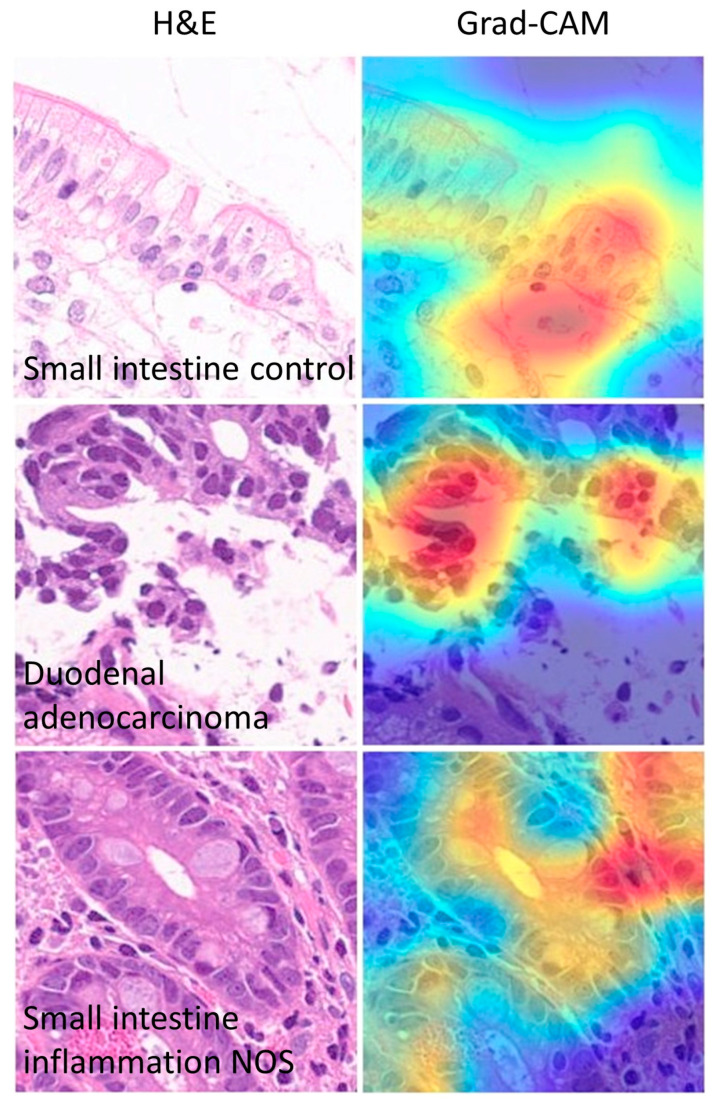
Other Grad-CAM images. The gradient-weighted class activation mapping (Grad-CAM) visualizes which parts of an image are important for the classification decision by the CNN. In this example, the most important part of the image for classification is the epithelial layer.

The Grad-CAM analysis visualized which parts of an image were important to the classification decision of the ResNet-18 network. From the histopathological point of view, the Grad-CAM analysis showed that the CNN was focusing on important parts of the tissue such as the epithelial layer and inflammation (Figure 15 and Figure 16).

**Figure 17:**
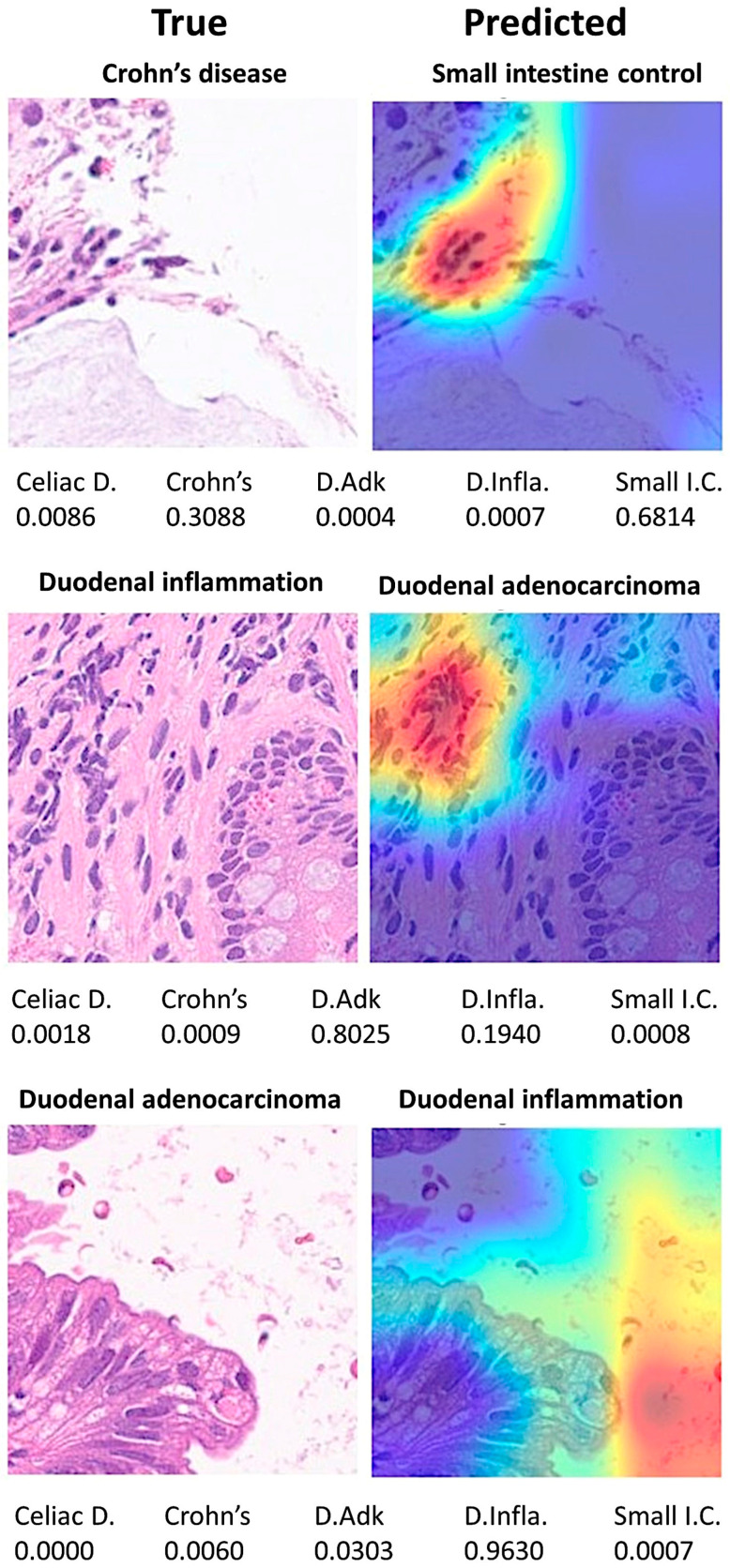
Grad-CAM analysis of discordant cases. The Grad-CAM visualizes which parts of an image are important to the classification decision of a network. Differences were due to images that did not have a clear diagnosis from a histological point of view and/or wrong focus area that was important to the classification of the network. Celiac D. (celiac Disease), Crohn’s (Crohn’s disease), D.Adk (duodenal adenocarcinoma), D.Infla. (duodenal inflammation), Small I.C. (small intestine control).

Discordant cases between true and predicted classes were also recorded and analyzed. In summary, differences were due to images that did not have a clear diagnosis from a histological point of view and/or wrong focus area that was important for the classification by the CNN (Figure 17).

The Grad-CAM technique utilizes the gradients of the classification score with respect to the final convolutional feature map, to identify the parts of an input image that most impact the classification score. The places where this gradient is large are exactly the places where the final score depends most on the data [61].

## 4. Discussion

Within the specialty of computer science, computer vision is a technique that allows computers to recognize the observable world. In the field of artificial intelligence, there are several machine learning and deep learning algorithms that build models that make predictions from images or videos [62]. Convolution neural networks are a type of supervised deep learning algorithm that are used for image recognition. A simple convolutional network comprises several steps, including image channel, convolutions, pooling, convolutions, pooling, flattening, artificial neural network full connection, and prediction [62].

The ResNet-18 network was used in this study. This convolutional neural network was a pre-trained model trained on a subset of the ImageNet database. The network is trained with more than a million images and managed to classify them into 1000 different categories [35]. In the medical field, this network has been used in several studies based on transfer learning, such as in the diagnosis of intracranial hemorrhage in CT scans [63], heartbeat classification of electrocardiogram (ECG) signals [64], dynamic gesture recognition [65], selective transplanting of leafy vegetable seedlings [66], automatic classification of malaria parasites on the blood smear [67], prostate imaging [68], classification of Alzheimer’s disease levels [69], and diabetic retinopathy [70], among others. Therefore, the ResNet-18 model is a useful network that can be applied to many types of studies, including our study of celiac disease.

Convolutional neural networks and image recognition have also been applied to celiac disease research, including the analysis of whole slide images [29,71,72,73,74] and endoscopic images [75,76]. Therefore, computer vision is a useful tool in the field of histopathology.

Our group has published several papers on the use of artificial intelligence, including machine learning and artificial neural networks, in the field of lymphoma research [77,78,79,80,81,82,83]. In these publications, the focus was on data analysis of gene expression levels in the context of immuno-oncology in lymphoma and other hematological neoplasia [77,78,79,80,81,82,83]. The most frequent lymphoma subtype that we analyzed was diffuse large b-cell lymphoma [78,79,80,81], which is one of the most frequent non-Hodgkin lymphomas [26]. In addition, we have also published data analysis-based studies on celiac disease in which we highlighted the importance of the B and T lymphocyte associated (BTLA) gene [21], and programmed cell death 1 ligand 1 (CD274 antigen) in ulcerative colitis [84]. The subject of this article represented a switch from data analytics to computer vision.

In this study, a confusion matrix was used to measure the performance of the trained network. The data (images) were split into three sets: a training set used for training (i.e., teaching) the network (70%), a validation set used for testing its performance as it was trained (10%), and a test set used after training to assess how well the network performed on new data (20%). The order of the images was randomized to ensure that the network learned the classes at a more even rate. In the Results Section, the confusion matrices of the test set were shown. Of note, if the data were imbalanced, the performance checking by accuracy could be deceptive. The confusion matrices of our study combined output data that was binary (Figure 9) and multiclass (Figure 10 and Figure 11). All performance parameters were high, including accuracy (defined as the proportion of correct predictions), precision (used in information retrieval, pattern recognition), recall (what in medicine is called sensitivity), and F1-score (measures test of accuracy). The fundamentals of clinical data science and modeling methodology are well described in chapter 8 of the book written by Frank J.W.M. Dankers et al. [85].

This study focused on the identification and classification of celiac disease images compared with normal small intestine images obtained from the duodenum and ileum. The accuracy of the network was very high. The model could handle and properly classify 3 classes with the addition of non-specific acute and chronic duodenal inflammation. Interestingly, when the 3 classes’ trained network was tested with duodenal adenocarcinoma, the network failed to realize that those samples were a different type of disease. Therefore, the use of automated computer vision analysis for the evaluation of histopathological slides is not recommended without the supervision of a pathology specialist. However, when the network was trained with 4 classes of histological subtypes, the network managed to differentiate celiac disease, duodenal inflammation, small intestine control, and duodenal adenocarcinoma with good performance, proving the usefulness of the convolutional neural network for classifying histological images.

To expand to other intestine inflammatory conditions, images of Crohn’s disease were included in the study, and the neural networks also manage to classify the different diseases properly.

There are several reports of artificial intelligence studies of celiac disease diagnosis and classification using both endoscopic and histological images.

Regarding endoscopic images, Ciaccio EJ et al. used videocapsule endoscopy images to detect pathologic alterations of 13 celiac and 13 control patients using a masking strategy that allowed nearly 80% accuracy [86]. Bing Nan Li implemented a principal component analysis (PCA) on videocapsule endoscopy images to develop a computerized tool of celiac disease recognition; a dataset of 240 images was used and the strip PCA method had an average recognition accuracy of 93.9% [87]. Jahmunah Vicnesh et al. used DAISY descriptors for the automated diagnosis of celiac disease by videocapsule endoscopy reaching an accuracy of 90% [88].

Regarding histological images, Joseph DiPalma et al. proposed a deep learning-based methodology for improving the computational efficiency of histology image classification based on distillation and self-supervision [89]. Florentino Luciano Caetano Dos Santos et al. used machine learning to assess and classify images of IgA-class endomysial autoantibody (EmA) images with an accuracy of 97% [90]. Kamran Kowsari et al. used a deep learning Hierarchical Medical Image classification (HMIC) approach to classify between celiac disease, environmental enteropathy, and histologically normal controls and the precision was 91% [91]. Joel En Wei Koh et al. used a Steerable Pyramid Transform (SPT) method and nonlinear features to automatically detect and classify celiac disease biopsy H&E images with an 89% accuracy [92]. Oliver Faust et al. used high-magnification biopsy images and a Support Vector Machine (SVM) to classify celiac disease versus normal control with an accuracy of 98% [30]. In the study of Prasenjit Das et al., the quantitative histological classification system based on software from Media Cybernetics and a Q-histological assessment reached a sensitivity of 94% [93]. J Denholm et al. classified H&E celiac disease and normal images from biopsies from several different types of digital image slide scanners. The workflow included patch extraction, stain normalization, and classification using ResNet50, and the final model reached an accuracy of 97%. Overall, these studies classified celiac disease images successfully but there is variability in methodology and accuracy. Our study is comparable to the one by J Denholm. However, our study included more different types of diagnosis.

## 5. Conclusions

In conclusion, a convolutional neural network based on the transfer learning of ResNet-18 was able to classify celiac disease, other duodenal pathological diseases, and tissue control with good performance. The current methodology is simple and reliable enough to be used as a real application. Narrow artificial intelligence (AI) is designed to perform tasks that typically require human intelligence, it operates within limited constraints and is task-specific. Therefore, all computer vision-based automated diagnoses should be supervised and validated by pathology medical specialists to identify other pathologies for which the network has not previously trained.

## Figures and Tables

**Table 1:** Performance parameters of the classification into 2 classes.

Class	Accuracy (%)	Precision (%)	Recall (%)	F1-Score (%)	Specificity (%)	False Positive Rate (%)
Celiac disease	99.97	99.93	100	99.97	99.96	0.04
Small intestine control	99.97	100	99.96	99.96	100	0

Recall is also referred to sensitivity and true positive rate (TPR). False positive rate (FPR).

**Table 2:** Performance parameters of the models for classification of celiac disease versus small intestine control.

Class	Accuracy (%)	Precision (%)	Recall (%)	F1-Score (%)	Specificity (%)	False Positive Rate (%)
ResNet-18	99.69	99.73	99.05	99.39	99.91	0.09
GoogLeNet	99.13	99.73	98.05	98.88	99.83	0.17
VGG-19	99.13	99.45	98.31	98.88	99.65	0.35
EfficientNet	99.42	99.79	98.71	99.25	99.87	0.13
MobileNet	99.02	98.97	98.50	98.74	99.35	0.65
NASNet	98.07	97.19	97.79	97.49	98.25	1.75

Recall is also referred to sensitivity and true positive rate (TPR). False positive rate (FPR).

**Table 3:** Performance parameters of the classification into 3 classes.

Class	Accuracy (%)	Precision (%)	Recall (%)	F1-Score (%)	Specificity (%)	False Positive Rate (%)
Celiac disease	99.68	99.59	99.32	99.45	99.83	0.17
Duodenal inflammation	98.37	96.48	96.72	96.60	98.89	1.11
Small intestine control	98.41	98.28	98.32	98.30	98.49	1.51

Recall is also referred as sensitivity and the true positive rate (TPR). False positive rate (FPR).

**Table 4:** Performance parameters of the classification into 4 classes.

Class	Accuracy (%)	Precision (%)	Recall (%)	F1-Score (%)	Specificity (%)	False Positive Rate (%)
Celiac disease	99.69	99.73	99.05	99.39	99.91	0.09
Duodenal adenocarcinoma	97.41	85.64	93.96	89.61	97.88	2.12
Duodenal inflammation	96.42	94.05	89.33	91.63	98.41	1.59
Small intestine control	98.38	97.85	98.15	98.00	98.53	1.47

Recall is also referred to sensitivity and true positive rate (TPR). False positive rate (FPR).

**Table 5:** Performance parameters of the classification into 4 classes.

Class	Accuracy (%)	Precision (%)	Recall (%)	F1-Score (%)	Specificity (%)	False Positive Rate (%)
Celiac disease	99.89	99.93	99.45	99.69	99.99	0.01
Crohn’s disease	98.51	97.20	98.03	97.61	98.73	1.27
Duodenal adenocarcinoma	98.94	91.67	96.33	93.94	99.19	0.81
Duodenal inflammation	98.20	96.40	91.49	93.88	99.39	0.61
Small intestine control	98.21	96.52	97.06	96.79	98.65	1.35

Recall is also referred to sensitivity and true positive rate (TPR). False positive rate (FPR).

**Figure A1:**
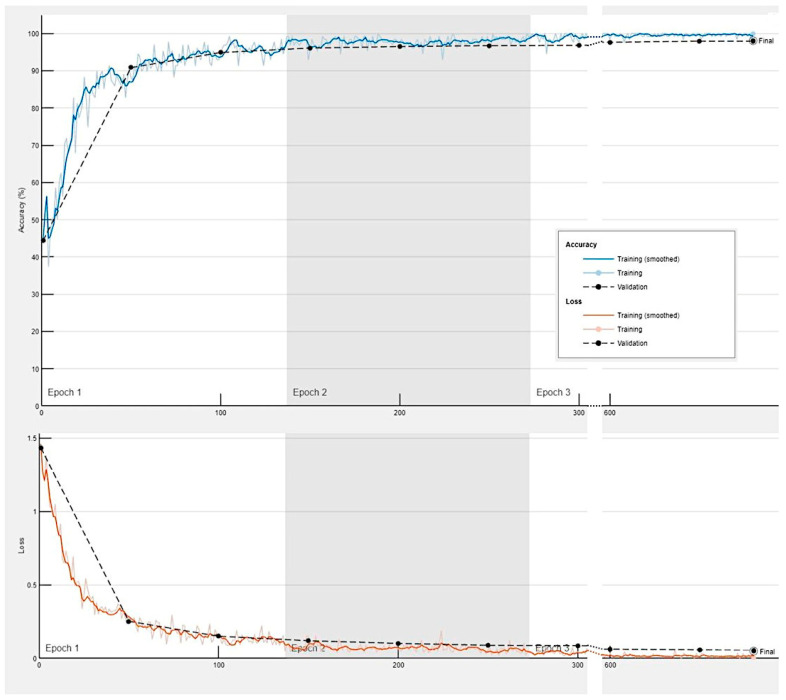
Training progress of the convolutional neural network for the classification of celiac disease, small intestine control, and duodenal inflammation.

**Figure A2:**
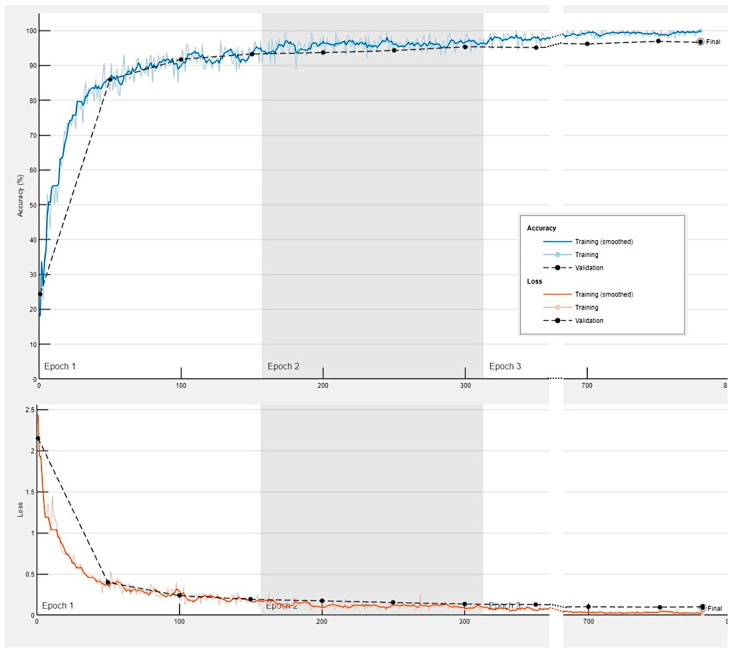
Training progress of the convolutional neural network for the classification of celiac disease, small intestine control, duodenal inflammation, and duodenal adenocarcinoma.

## Data Availability

All the data, including methodology, are available upon reasonable request to Dr. Joaquim Carreras (joaquim.carreras@tokai-u.jp).

## Supplementary Materials

The following supporting information can be downloaded at: https://www.mdpi.com/article/10.3390/jimaging10080200/s1, Supplementary_Output_5_classes.

## Appendix A

**Table A1:** Clinicopathological characteristics of celiac disease cases.

Age	Sex	Biopsy Location	Diagnosis	Marsh–Oberhuber Classification
70	Male	Duodenum	Celiac Disease	3a
62	Male	Pylorus/duodenum	Celiac Disease/Chronic gastritis	2
62	Male	Duodenum	Celiac Disease	2
78	Female	Duodenum	Celiac Disease	3b
59	Male	Duodenum	Celiac Disease	3a
44	Female	Duodenum	Celiac Disease	2
17	Female	Duodenum	Celiac Disease	3b
56	Female	Duodenum	Celiac Disease	3a
54	Female	Duodenum	Celiac Disease	2
58	Female	Duodenum	Celiac Disease	3b
61	Female	Duodenum	Celiac Disease	3c
45	Male	Duodenum	Celiac Disease	3a
70	Female	Duodenum	Celiac Disease	2
40	Female	Duodenum	Celiac Disease	3a
61	Female	Duodenum	Celiac Disease	3c
44	Female	Duodenum	Celiac Disease	3a

## Appendix B

**Table A2:** Design of trained network.

Trained Network		Trained Network Connections	
Num.	Layers	1 Source	2 Destination
1	1 × 1 ImageInputLayer	‘data’	‘conv1’
2	1 × 1 Convolution2DLayer	‘conv1’	‘bn_conv1’
3	1 × 1 BatchNormalizationLayer	‘bn_conv1’	‘conv1_relu’
4	1 × 1 ReLULayer	‘conv1_relu’	‘pool1’
5	1 × 1 MaxPooling2DLayer	‘pool1’	‘res2a_branch2a’
6	1 × 1 Convolution2DLayer	‘pool1’	‘res2a/in2’
7	1 × 1 BatchNormalizationLayer	‘res2a_branch2a’	‘bn2a_branch2a’
8	1 × 1 ReLULayer	‘bn2a_branch2a’	‘res2a_branch2a_relu’
9	1 × 1 Convolution2DLayer	‘res2a_branch2a_relu’	‘res2a_branch2b’
10	1 × 1 BatchNormalizationLayer	‘res2a_branch2b’	‘bn2a_branch2b’
11	1 × 1 AdditionLayer	‘bn2a_branch2b’	‘res2a/in1’
12	1 × 1 ReLULayer	‘res2a’	‘res2a_relu’
13	1 × 1 Convolution2DLayer	‘res2a_relu’	‘res2b_branch2a’
14	1 × 1 BatchNormalizationLayer	‘res2a_relu’	‘res2b/in2’
15	1 × 1 ReLULayer	‘res2b_branch2a’	‘bn2b_branch2a’
16	1 × 1 Convolution2DLayer	‘bn2b_branch2a’	‘res2b_branch2a_relu’
17	1 × 1 BatchNormalizationLayer	‘res2b_branch2a_relu’	‘res2b_branch2b’
18	1 × 1 AdditionLayer	‘res2b_branch2b’	‘bn2b_branch2b’
19	1 × 1 ReLULayer	‘bn2b_branch2b’	‘res2b/in1’
20	1 × 1 Convolution2DLayer	‘res2b’	‘res2b_relu’
21	1 × 1 BatchNormalizationLayer	‘res2b_relu’	‘res3a_branch2a’
22	1 × 1 ReLULayer	‘res2b_relu’	‘res3a_branch1’
23	1 × 1 Convolution2DLayer	‘res3a_branch2a’	‘bn3a_branch2a’
24	1 × 1 BatchNormalizationLayer	‘bn3a_branch2a’	‘res3a_branch2a_relu’
25	1 × 1 Convolution2DLayer	‘res3a_branch2a_relu’	‘res3a_branch2b’
26	1 × 1 BatchNormalizationLayer	‘res3a_branch2b’	‘bn3a_branch2b’
27	1 × 1 AdditionLayer	‘bn3a_branch2b’	‘res3a/in1’
28	1 × 1 ReLULayer	‘res3a_branch1’	‘bn3a_branch1’
29	1 × 1 Convolution2DLayer	‘bn3a_branch1’	‘res3a/in2’
30	1 × 1 BatchNormalizationLayer	‘res3a’	‘res3a_relu’
31	1 × 1 ReLULayer	‘res3a_relu’	‘res3b_branch2a’
32	1 × 1 Convolution2DLayer	‘res3a_relu’	‘res3b/in2’
33	1 × 1 BatchNormalizationLayer	‘res3b_branch2a’	‘bn3b_branch2a’
34	1 × 1 AdditionLayer	‘bn3b_branch2a’	‘res3b_branch2a_relu’
35	1 × 1 ReLULayer	‘res3b_branch2a_relu’	‘res3b_branch2b’
36	1 × 1 Convolution2DLayer	‘res3b_branch2b’	‘bn3b_branch2b’
37	1 × 1 BatchNormalizationLayer	‘bn3b_branch2b’	‘res3b/in1’
38	1 × 1 ReLULayer	‘res3b’	‘res3b_relu’
39	1 × 1 Convolution2DLayer	‘res3b_relu’	‘res4a_branch2a’
40	1 × 1 BatchNormalizationLayer	‘res3b_relu’	‘res4a_branch1’
41	1 × 1 Convolution2DLayer	‘res4a_branch2a’	‘bn4a_branch2a’
42	1 × 1 BatchNormalizationLayer	‘bn4a_branch2a’	‘res4a_branch2a_relu’
43	1 × 1 AdditionLayer	‘res4a_branch2a_relu’	‘res4a_branch2b’
44	1 × 1 ReLULayer	‘res4a_branch2b’	‘bn4a_branch2b’
45	1 × 1 Convolution2DLayer	‘bn4a_branch2b’	‘res4a/in1’
46	1 × 1 BatchNormalizationLayer	‘res4a_branch1’	‘bn4a_branch1’
47	1 × 1 ReLULayer	‘bn4a_branch1’	‘res4a/in2’
48	1 × 1 Convolution2DLayer	‘res4a’	‘res4a_relu’
49	1 × 1 BatchNormalizationLayer	‘res4a_relu’	‘res4b_branch2a’
50	1 × 1 AdditionLayer	‘res4a_relu’	‘res4b/in2’
51	1 × 1 ReLULayer	‘res4b_branch2a’	‘bn4b_branch2a’
52	1 × 1 Convolution2DLayer	‘bn4b_branch2a’	‘res4b_branch2a_relu’
53	1 × 1 BatchNormalizationLayer	‘res4b_branch2a_relu’	‘res4b_branch2b’
54	1 × 1 ReLULayer	‘res4b_branch2b’	‘bn4b_branch2b’
55	1 × 1 Convolution2DLayer	‘bn4b_branch2b’	‘res4b/in1’
56	1 × 1 BatchNormalizationLayer	‘res4b’	‘res4b_relu’
57	1 × 1 Convolution2DLayer	‘res4b_relu’	‘res5a_branch2a’
58	1 × 1 BatchNormalizationLayer	‘res4b_relu’	‘res5a_branch1’
59	1 × 1 AdditionLayer	‘res5a_branch2a’	‘bn5a_branch2a’
60	1 × 1 ReLULayer	‘bn5a_branch2a’	‘res5a_branch2a_relu’
61	1 × 1 Convolution2DLayer	‘res5a_branch2a_relu’	‘res5a_branch2b’
62	1 × 1 BatchNormalizationLayer	‘res5a_branch2b’	‘bn5a_branch2b’
63	1 × 1 ReLULayer	‘bn5a_branch2b’	‘res5a/in1’
64	1 × 1 Convolution2DLayer	‘res5a_branch1’	‘bn5a_branch1’
65	1 × 1 BatchNormalizationLayer	‘bn5a_branch1’	‘res5a/in2’
66	1 × 1 AdditionLayer	‘res5a’	‘res5a_relu’
67	1 × 1 ReLULayer	‘res5a_relu’	‘res5b_branch2a’
68	1 × 1 GlobalAveragePooling2DLayer	‘res5a_relu’	‘res5b/in2’
69	1 × 1 FullyConnectedLayer	‘res5b_branch2a’	‘bn5b_branch2a’
70	1 × 1 SoftmaxLayer	‘bn5b_branch2a’	‘res5b_branch2a_relu’
71	1 × 1 ClassificationOutputLayer	‘res5b_branch2a_relu’	‘res5b_branch2b’
		‘res5b_branch2b’	‘bn5b_branch2b’
		‘bn5b_branch2b’	‘res5b/in1’
		‘res5b’	‘res5b_relu’
		‘res5b_relu’	‘pool5’
		‘pool5’	‘fc’
		‘fc’	‘prob’
		‘prob’	‘classoutput’
